# Using an Aqueous Suspension of *Duddingtonia flagrans* Chlamydospores and a Hexane Extract of *Artemisia cina* as Sustainable Methods to Reduce the Fecal Egg Count and Larvae of *Haemonchus contortus* in the Feces of Periparturient Ewes

**DOI:** 10.3390/pathogens14020105

**Published:** 2025-01-21

**Authors:** Héctor Alejandro de la Crúz-Crúz, Rosa Isabel Higuera-Piedrahita, Alejandro Zamilpa, Yazmín Alcalá-Canto, Ana Yuridia Ocampo-Gutiérrez, Luis David Arango-de la Pava, María Eugenia López-Arellano, Daniel Hernandez-Patlan, Jorge Alfredo Cuéllar-Ordaz, Pedro Mendoza-de Gives

**Affiliations:** 1 Laboratory of Helminthology, National Centre for Disciplinary Research in Animal Health and Innocuity (CENID-SAI), National Institute for Research in Forestry, Agriculture, and Livestock, INIFAP-SADER, Jiutepec 62550, Mexico; delacruz@unam.mx (H.A.d.l.C.-C.); yumiga2002@yahoo.com.mx (A.Y.O.-G.); mlopez.arellano@gmail.com (M.E.L.-A.); 2 Laboratory 3, Multidisciplinary Research Unit, Superior Studies Faculty at Cuautitlán, National Autonomous University of Mexico (FESC-UNAM), Cuautitlán Izcalli 54714, Mexico; rhiguera05@comunidad.unam.mx (R.I.H.-P.); luis.david@cuautitlan.unam.mx (L.D.A.-d.l.P.); jcuellar@unam.mx (J.A.C.-O.); 3 Master’s and Doctorate Program in Health Sciences and Animal Production, Faculty of Veterinary Medicine and Animal Husbandry, National Autonomous University of Mexico, Ciudad de México 04510, Mexico; 4 Southern Biomedical Research Center, Mexican Social Security Institute (CIBIS-IMSS), Argentina 1, Col. Centro, Xochitepec 62790, Mexico; azamilpa_2000@yahoo.com.mx; 5 Faculty of Veterinary Medicine and Zootechnics, National Autonomous University of Mexico, Ciudad de México 04510, Mexico; yazmin@unam.mx; 6 Laboratory 5: LEDEFAR, Multidisciplinary Research Unit, Superior Studies Faculty at Cuautitlán, National Autonomous University of Mexico (FESC-UNAM), Cuautitlán Izcalli 54714, Mexico; danielpatlan@comunidad.unam.mx; 7 Nanotechnology Engineering Division, Polytechnic University of the Valley of Mexico, Tultitlán 54910, Mexico

**Keywords:** *Duddingtonia flagrans*, *Artemisia cina*, *Haemonchus contortus*, EPG reduction, larvae reduction

## Abstract

This study evaluated the effectiveness of *Duddingtonia flagrans* chlamydospores and an *Artemisia cina* hexane extract in reducing *Haemonchus contortus* fecal egg counts and larvae in periparturient ewes. This study involved five groups of four ewes: a control group, an ivermectin group, an *A. cina* oral extract group, a *D. flagrans* group, and a combined treatment group. Treatments began two weeks before delivery, with ivermectin administered 15 days before delivery. Fecal samples were collected every fifteen days to estimate parasite egg counts per gram of feces (EPG) and assess larvae reductions. The results showed very low EPG values for ivermectin and *D. flagrans* treatments (175 and 150, respectively). The control and combined treatment groups had EPG values rise to 3000 and 4100 by day 15. The EPG values for the *A. cina* group reached 850 and 533 in later samplings. Throughout the study, the *D. flagrans* and *A. cina* groups maintained low EPG values, with the highest recorded values being 50 and 0, respectively. All treatments significantly reduced the larvae in the fecal cultures: *D. flagrans* (97.4% reduction), ivermectin (91.4%), *Artemisia cina* (89.9%), and the combined treatment (84.3%).

## 1. Introduction

*Haemonchus contortus* is one of the most pathogenic parasitic nematodes affecting small ruminants worldwide. Haemonchosis causes clinical signs in the flocks that are characterized by different levels of anemia, weight loss, and diminishing meat and milk production [1]. In extreme cases, it can cause the death of the animals [2,3]. The high reproduction rate and the rapid spread of antihelmintic resistance in parasites against most commercially available antiparasitic chemical drugs make *H. contortus* a concerning problem for producers trying to work the issue out, increasing the number of chemical treatments required [4]. During the reproductive process in ewes, several critical physiological changes, including a hormonal disbalance, are responsible for severe stress that results in a temporal reduction in natural immunity [5]. Such a depression in the immune system is used by parasites, such as *H. contortus*, to take advantage of the low capability of the immunological system of animals to self-defend against pathogens, including parasites [5]. In this context, the *H. contortus* adult females increase their egg output, and the fecal egg count can reach high quantities, becoming a worrying problem with epidemiological consequences, spreading eggs, and contaminating the grazing lands. This phenomenon is called “spring rise” [6]. Prophylaxis for gastrointestinal nematode infections, including haemonchosis, is mainly based on the use of anthelmintic drugs [7]. However, this system has essential disadvantages, i.e., the imminent development of anthelmintic resistance in the parasites [7,8]. Another disadvantage of the use of these drugs is the potential risk to public health since drug residues can remain in milk, meat, or sub-products for human consumption [9]. Anthelmintics can also trigger an environmental problem since some chemical drugs administered to the animals are eliminated through feces or urine, affecting beneficial invertebrates such as fecal beetles [10] and the beneficial soil microbial biomass [11]. Nematophagous fungi are soil microorganisms that live as saprophytic microorganisms feeding on organic matter. However, they transform into predatory nematode fungi when they detect the presence of nematodes in their proximity [12]. *Duddingtonia flagrans* is a nematode-trapping fungus that captures nematodes by developing three-dimensional adhesive nets. Captured nematodes are penetrated by an infecting bulb (appressorium), and mycelia colonize the whole nematode body. Eventually, the fungus takes its nitrogen and carbon source from the nematode tissues [13]. This species is considered a potential biotechnological tool against parasitic nematodes affecting the gastrointestinal tract of ruminants [14]. Studies have revealed that the oral administration of an aqueous suspension of *D. flagrans* exerts a lethal effect against the larvae of gastrointestinal parasitic nematodes in sheep feces [15,16]. Another sustainable strategy to control gastrointestinal parasitic nematodes is using plants or their metabolites with nematocidal activity. *Artemisia cina*, also called Wormwood from the East, is a perennial herbaceous plant widely used in traditional medicine as a natural remedy because of its multiple medical properties. Studies have shown this plant contains bioactive nematocidal compounds such as sesquiterpenes, flavonoids, and other compounds against different nematodes, including *H. contortus* [17]. The objective of the present study is to assess the reduction in the *H. contortus* fecal egg count, as well as the infective larvae in fecal cultures of periparturient ewes treated with an oral aqueous suspension containing *D. flagrans* chlamydospores and an *A. cina* hexanic extract. 

## 2. Materials and Methods

### 2.1. Location

The taxonomical identification and the fungal production were performed at the Laboratory of Helminthology of the National Center of Research Disciplinary in Animal Health and Innocuity (CENID-SAI-INIFAP) in Jiutepec Municipality, Morelos State, Mexico. The animal selection, the artificial infection of ewes, the application of treatments, and the fecal sampling were performed at a commercial sheep farm, “Quinta Mejor”, in Zumpango Municipality, State of Mexico. Zumpango is located between parallels 19°43′ and 19°54′ north latitude; the meridians 98°58′ and 99°12′ west longitude; altitude between 2200 and 2800 m presents a temperature range of 14–16 °C, precipitation range 500–700 mm in semi-dry climate with rain in summer, with an average humidity of 47% in this season.

### 2.2. Biological Material

#### 2.2.1. Obtaining *Duddingtonia flagrans* Chlamydospores

An autochthonous strain of the fungus was initially obtained from the feces of a grazing lamb at Fierro del Toro Village, Huitzilac Municipality, Morelos State, Mexico [18]. The strain has been maintained under laboratory conditions by passing it to agar plates and storing it under cryopreservation conditions. A large amount of chlamydospores was achieved by cultivating the fungus on potato–dextrose agar plates at room temperature (18–25 °C) for 3 weeks before harvesting. After incubation, chlamydospores were separated from the mycelium by adding 5 mL of water and scraping the agar surface with a sterile slide. The recovered liquid containing chlamydospores was stored in 1 L volume crystal containers (Kimax, labs, Mexico). Chlamydospores were quantified using the Neubauer chamber technique. A Neubauer hemocytometer was employed by adding nine microliters of the chlamydospore suspension into each of the two counting chambers. The chamber was then covered with a coverslip, and the spores were observed under a microscope. Chlamydospores were counted in the four corner squares, excluding those touching the top and left borders. The concentration of chlamydospores was estimated based on the average number counted in these four squares. Finally, the concentration was adjusted according to the dilution of the suspension [19].

#### 2.2.2. Obtaining Infective *Haemonchus contortus* Larvae (L_3_)

An *H. contortus* strain (FES-Cuautitlán strain) was initially obtained from the feces of a naturally infected hair lamb at “La Milpa” farm, situated in Jilotepec Municipality, State of Mexico. The population of this nematode has been maintained by cryopreservation and through passes in nematode-free lambs [20]. For the current experiment, a significant quantity of infective *H. contortus* larvae was obtained by orally infecting a 3-month-old Creole male lamb with a single dose of 10 mL of an aqueous suspension containing 5000 infective *H. contortus* larvae. After 20 days post-infection (pre-patent period), fresh feces containing eggs of the parasite were directly collected from the rectum of this animal and macerated using a mortar and a pestle and processed by growing fecal cultures in Petri dishes supplemented with a film of water following the modified Corticelli–Lay technique [21,22] and maintained at room temperature (18–25 °C). These conditions help promote eggs’ optimum development until they reach the third infective stage of the parasite. The egg-donor lamb was strictly maintained under controlled conditions according to principles of animal welfare and the elimination of unnecessary animal suffering according to Norma Oficial Mexicana (official rule number) NOM-052-ZOO-1995 (https://www.gob.mx/senasicaas, accessed on 10 December 2024) as well as the Ley Federal de Sanidad Animal (Federal Law for Animal Health) DOF 07-06-2012 (http://www.diputados.gob.mx/LeyesBiblio/ref/lfsa.htm, accessed on 10 December 2024) and DOF 07-06-2012, established at the FES-Cuautitlán, UNAM.

#### 2.2.3. Obtaining *Artemisia cina* n-Hexane Extract

Two hundred grams of dried *A. cina* (Asteraceae) leaves and stems collected at their pre-flowering stage were obtained from a commercial greenhouse (Hunab Laboratory, Mexico City, Mexico). Plant material was macerated using n-hexane for 48 h at room temperature (23–25 °C) [23]. The macerated extract was filtered using a Whatman paper No. 4, and the solvent was removed by low-pressure distillation using a rotary evaporator (Heidolph Laborota 4000, Heidolph Instruments, Schwabach, Germany) and the resulting material was eventually lyophilized and kept at 4 °C until use. 

### 2.3. Animals

Twenty Romanov x East Friesian crossbred, aged from one to two years old, periparturient ewes, were kept under housing conditions at an experimental unit in Zumpango Municipality, State of Mexico. This flock was maintained under controlled reproductive management of estrus synchronization using intravaginal sponges impregnated with flurogesterone acetate (Chronogest CR^®^, MSD lab, Rahway, NJ, USA). This product is a progestogen called Chronolone. Twenty milligrams of this product remained for 12 days before controlled breeding. Thirty days after breeding and pregnancy, ewes were diagnosed using ultrasound equipment (KX2600, Ultrasonic Diagnostic Instruments, Xuzhou, China) to eventually achieve a homogeneous group of pregnant ewes at birth time. One month before starting the treatments, the whole experimental flock was orally dewormed with Albendazole (Koptisin ovine, Chinoin lab, Mexico City, Mexico) at 10 µg/kg BW. One month after the anthelmintic treatments, the whole flock was orally infected with 12 mL of an aqueous suspension containing 5000 infective *H. contortus* larvae per animal using a rubber esophageal tube. Five weeks after induced parasitic infection (30 days before births), fecal samples for every ewe were taken directly from the rectum to perform the McMaster technique. Samples were taken every 15 days to draw a dynamic picture of fecal egg elimination in every experimental group. 

### 2.4. Experimental Groups

Five groups of four ewes each were randomly distributed according to the following experimental design: Group (1) untreated group (control), Group (2) Treated with a single subcutaneous injection of Ivermectin at 200 µg/kg of Body Weight (BW) (chemical treatment) fifteen days before delivery, Group (3) Treated twice with an oral hexanic extract of *A. cina* at 4 mg/kg BW (15 days before delivery and 15 days after delivery), Group (4) Orally treated with *D. flagrans* at a dose of 1 × 10^6^ chlamydospores per kg of BW every third day from 15 days before delivery until day 42 (fungal treatment), and Group (5) Treated with *D. flagrans* chlamydospores + *A. cina* extract at same doses and times of applications as Group 4 (combined treatment). 

Every fifteen days, the whole flock was sampled to collect feces directly from the rectum, and the McMaster technique was performed to estimate the number of parasite eggs eliminated per gram of feces (EPG). Feces were also used to prepare fecal cultures to obtain and assess the infective larvae population in feces. The means of EPG and larvae recovered from the feces of animals during the whole experiment were compared among the groups. The means of EPG and larvae in control groups were considered 100% (without the effects of fungi, plant extracts, or anthelmintics). The results were expressed as eggs or larvae reduction percentages based on the following formula:

$$Reduction\%=\frac{\bar{X}Control-\bar{X}Treatment}{\bar{X}Control}\times 100$$

where

$\bar{X}$Control = Mean of eggs or larvae recovered from control group

$\bar{X}$Treatment = Mean of eggs or larvae recovered from the treated group

### 2.5. Statistical Analysis

The nonparametric Kruskal–Wallis test was performed to analyze the mean number of eggs and larvae from *H. contortus* in feces (EPG), followed by a post-hoc analysis using Dunn’s multiple comparisons test to assess differences (*p* < 0.05). Data from both studies were expressed as median (Max,Min). GraphPad Prism version 10.4.1 software (GraphPad Software, San Diego, CA, USA) was used for statistical analyses and figure preparation. The assumptions of normality and homogeneity of variances, using the Shapiro–Wilk test and the Leven test at 95% confidence, were met for each study variable.

## 3. Results

### 3.1. Reduction in Haemonchus contortus Egg Population Recovered from Feces Attributed to Effect of Different Treatments

During all the samplings, the EPG values recorded for the chemical treatment (ivermectin) and the fungal treatment (*D. flagrans*) were very low, reaching the highest EPG values of 175 and 150, respectively, on day 15. After we compared these records with those obtained in the control group (1129), it led to essential reduction percentages of 93 and 89% in the EPG for ivermectin and *D. flagrans*, respectively. From the births (3rd sampling), the values in the control and combined groups (fungi + plant extract) increased to 3000 and 4100 EPG values on the 15th day. Likewise, *A. cina* increased to reach 850 and 533 EPG during the last two samplings (15 and 30 days after births). It is interesting to remark on the fact that from the first EPG record along with all the samples (from −30 days before delivery to +30 days after delivery), the EPG values recorded in both the *D. flagrans* and *A. cina* groups remained very low, within a range of 50–150 for *D. flagrans* and 25–875 for *A. cina*. The EPG values obtained from the treatments recorded during the whole experiment are summarized in Table 1. 

### 3.2. Reduction in Infective Haemonchus contortus Larvae Population (L_3_) Recovered from Feces Attributed to Effect of Different Treatments

Table 2 shows the results of this experiment expressed as the means of recovered larvae, reduction percentages, and overall mean of fecal larval reduction. The four assessed treatments, fungi, plant extract, fungi + plant extract, and ivermectin, showed very low means of recovered larvae during samplings compared with the control group (with no treatment). The four treatments showed essential reductions in the means of larvae recovered from fecal cultures, with the highest reductions in the groups treated with the following overall larval reductions: *D. flagrans* (97.4%), followed by Ivermectin (91.4%), *A. cina* (89.9%) and the combination of *D. flagrans* + *A. cina* extract (84.3%). 

## 4. Discussion

### 4.1. Reduction in Haemonchus contortus Egg Population Recovered from Feces Attributed to Effect of Different Treatments

The results of the EPG values in the different samplings from the experiment show that both treatments, the oral administration of a *D. flagrans* chlamydospore suspension as well as the oral administration of a hexanic extract of *A. cina*, resulted in a very high efficacy in reducing the fecal counts of eggs per gram of feces in periparturient ewes when both treatments were employed, with individual values being 90% for *D. flagrans* and 70% for *A. cina*. In contrast, the EPG recorded in the control group increased around birth, reaching values close to 3000 EPG on the 4th sampling day (15 days post-birth). This increase in the EPG values in periparturient ewes was expected since the immune response is dramatically reduced due to the enormous physiological and hormonal pressure during this reproductive stay, particularly regarding prolactin and cortisol. This condition allows the *H. contortus* adult females living in the abomasum to produce large amounts of eggs to be eliminated into the environment to continue their development on the field; this phenomenon is known as the post-parturient rise [24]. It is important to note that the post-partum increase in the elimination of parasite eggs is significant from an epidemiological perspective. Many parasitic eggs can spread through extensive grazing areas, contaminating other animals in the flock [25,26]. Likewise, due to the dramatic physiological and hormonal stress resulting from the peripartum period and lambing, ewes should be subjected to a unique care program, particularly avoiding regular chemotherapy [27]. In this context, some anthelmintics, i.e., benzimidazoles, can be of risk for pregnant or lambing ewes [28]. In general, the adverse effects caused by anthelmintic treatments are due to the misuse of anthelmintic drugs, mainly when the dose recommended by the manufacturers is exceeded, which can cause teratogenic effects in sheep [29,30]. In this study, the oral administration of a hexanic extract from *A. cina* effectively reduced EPG values in periparturient sheep, so we assume that the secondary compounds present demonstrate an anthelmintic capacity, as reported in goats [31]. 

On the other hand, Table 3 presents additional studies showing the impact of chlamydospores on reducing gastrointestinal parasitic infective larvae counts in ruminants. 

The results in these studies have shown that the effect of the oral administration of different doses of *D. flagrans* chlamydospores, either in large or small ruminants, leads to significant reductions in the eggs and larvae of gastrointestinal parasitic nematodes, such as occurred in the present study. The control group was focused on a stratus of the flock, which is very important from an epidemiological point of view, since the period surrounding birth results in a significant increase in the dissemination of the eggs of the parasites by periparturient ewes to extensive grazing areas, increasing the contamination of the animals in the flocks. The use of alternative methods of control, either with nematophagous fungi or plant extracts, could act as a natural tool for control, avoiding the excessive use of anthelmintic drugs and improving the health and productivity of animals. 

It is worth highlighting the fact that the oral administration of the aqueous suspension of *D. flagrans* chlamydospores reduced the EPG counts, as it is well-known that the chlamydospores of this species of nematophagous fungi exert their predatory effects when they pass through the gastrointestinal tract of sheep and reach the feces. They germinate, colonize the feces, form trapping devices, and kill and feed on the larvae [34]. However, it is also well established that *D. flagrans* and other species of nematophagous fungi produce a wide variety of secondary metabolite-derived compounds and fungal enzymes with diverse biological activities, including nematicidal effects against parasites of importance in agriculture and the livestock industry [35,36,37]. So, it is probable that fungal metabolites with nematocidal activity could be released into the liquid cultures, and they could be responsible for the fecal EPG reduction rates. Regarding the combination of treatments using *D. flagrans* chlamydospores and the *A. cina* hexanic extract, the results showed an antagonistic effect between both treatments. Instead of increasing the efficacy of both individual therapies, the EPG values increased. The fact that this antagonistic effect was only observed in the EPG values and not in the larvae counts could be explained by the interactions of antagonistic metabolites produced by both the plant and the fungus, which may lead to this antagonistic effect. In contrast, this effect was not seen in the larval counts. After the animals consume the fungal chlamydospores, these spores are expelled in their feces, where they develop trapping devices that capture larvae in situ, ultimately leading to a reduction in the larval population [34].

### 4.2. Reduction in Infective H. contortus Larvae Population (L_3_) Recovered from Feces Attributed to the Effect of the Different Treatments

Regarding the reduction in the larvae population in fecal cultures from ewes of the different treatments, all the groups, including the *A. cina* plant extract, *D. flagrans* chlamydospores aqueous suspension, as well as the combination of both treatments, resulted in an excellent reduction in the population of infective *H. contortus* larvae. The highest efficacy of the treatments was reached with *D. flagrans*, followed by the plant extract and the combination of both strategies. Some records about the effect of the oral administration of Artemisia spp. extracts in sheep on the reduction in the eggs and infective larvae from gastrointestinal parasitic nematodes are shown in Table 4. 

Regarding the efficacy of the anthelmintic drug, it is interesting that the efficacy of ivermectin was not as we expected, since its overall efficacy reached only a 91.4% reduction in the larvae population, which could probably be due to an ivermectin anthelmintic resistance in the parasite. Interestingly, in an unpublished study conducted by our group, we identified the P-glycoprotein (P-gp) gene associated with ivermectin resistance in this strain of *H. contortus*. The findings from this research are vital for future studies aimed at exploring the potential use of *D. flagrans* chlamydospores and *A. cina* hexanic extracts in an integrated sustainable control program.

## 5. Conclusions 

The findings of this study indicate that administering an oral suspension of chlamydospores from the nematophagous fungus *D. flagrans*, or a hexane extract from the plant *A. cina*, significantly reduces the presence of *H. contortus* eggs and larvae in the feces of periparturient ewes. These sustainable control strategies offer considerable benefits from an epidemiological standpoint, particularly during the postpartum period when nematode eggs are widely dispersed in grazing areas. The adoption of alternative control methods, as evaluated in this study, can enhance environmental sustainability, improve flock health and productivity, and mitigate economic losses by reducing reliance on conventional anthelmintic drugs.

## Figures and Tables

**Table 1:** Means of EPG values recorded from fecal samplings of five groups of pre-parturient ewes treated with ivermectin, *Duddingtonia flagrans* chlamydospores, *Artemisia cina* hexanic extract, and a combined treatment (fungi + plant extract).

Day of Sampling		Control	Iv	*Df*	*Ac*	*Df*/*Ac*
**Prepartum**						
d −30	Mean ± SE	0.0 ± 0.0	0.0 ± 0.0	137.5 ± 37.5	0.0 ± 0.0	0.0 ± 0.0
	Median (Min–Max)	0.0 (0–0) ^a^	0.0 (0–0) ^a^	150.0 (50–200) ^b^	0.0 (0–0) ^a^	0.0 (0–0) ^a^
d −15	Mean ± SE	133.3 ± 44.1	175.0 ± 43.3	150.0 ± 84.2	25.0 ± 14.4	50.0 ± 20.4
	Median (Min–Max)	150.0 (50–200)	150.0 (100–300)	75.0 (50–400)	25.0 (0–50)	50.0 (0–100)
**Partum**						
d 0	Mean ± SE	641.7 ± 282.2	62.5 ± 47.3	137.5 ± 55.4	325.0 ± 118.1	662.5 ± 498.5
	Median (Min–Max)	600.0 (175–1150)	25.0 (0–200)	100.0 (50–300)	400.0 (0–500)	225.0 (50–2150)
**Postpartum**						
d +15	Mean ± SE	2800.0 ± 1311.5	62.5 ± 62.5	125.0 ± 32.3	875.0 ± 340.6	4287.5 ± 967.3
	Median (Min–Max)	3800.0 (200–3800) ^ab^	0.0 (0–250) ^b^	125.0 (50–200) ^ab^	975.0 (0–1550) ^ab^	3725.0 (2650–7050) ^a^
d +30	Mean ± SE	2883.3 ± 1728.3	75.0 ± 43.3	150.0 ± 54.0	533.3 ± 533.3	2562.5 ± 678.7
	Median (Min–Max)	2500.0 (100–6050)	75.0 (0–150)	125.0 (50–300)	0.0 (0–1600)	2950.0 (1550–4500)

Superscript letters (^a,b^) between columns indicate significant differences among groups at *p* < 0.05. Iv = ivermectin, *Df* = *Duddingtonia flagrans*, *Ac* = *Artemisia cina*, and *Df*/*Ac* = combination of *D. flagrans* with *A. cina*.

**Table 2:** Means of *Haemonchus contortus* larvae recovered from fecal cultures of artificially infected ewes under four control strategies (before, during, and postpartum) and overall reduction percentages.

Day of Sampling		Control	Iv	*Df*	*Ac*	*Df*/*Ac*
**Prepartum**						
d −30	Mean ± SE	0.0 ± 0.0	0.0 ± 0.0	0.0 ± 0.0	0.0 ± 0.0	0.0 ± 0.0
	Median (Min–Max)	0.0 (0–0)	0.0 (0–0)	0.0 (0–0)	0.0 (0–0)	0.0 (0–0)
d −15	Mean ± SE	500.0 ± 173.2	942.5 ± 466.8	25.0 ± 25.0	120.0 ± 120.0	240.0 ± 240.0
	Median (Min–Max)	500.0 (200–800)	845.0 (0–2080)	0.0 (0–100)	0.0 (0–480)	0.0 (0–960)
**Partum**						
d 0	Mean ± SE	4683.3 ± 4460.23	906.5 ± 482.2	100.0 ± 100.0	435.8 ± 105.4	932.5 ± 326.0
	Median (Min–Max)	450.0 (0–13,600)	773.0 (0–2080)	100.0 (0–400)	478.5 (148–638)	1138.0 (1–1453)
**Postpartum**						
d +15	Mean ± SE	9901.7 ± 7853.5	17.0 ± 17.0	125.0 ± 75.0	542.3 ± 257.0	233.0 ± 222.45
	Median (Min–Max)	2505.0 (1600–25,600) ^a^	0.0 (0–68.0) ^b^	100.0 (0–300.0) ^ab^	394.0 (105–1276) ^ab^	16.0 (0–900.0) ^ab^
d +30	Mean ± SE	4816.67 ± 4592.2	100.0 ± 57.735	100.0 ± 100.0	137.5 ± 121.4	225.0 ± 225.0
	Median (Min–Max)	350.0 (0–13,600)	100.0 (0–200.0)	0.0 (0–400.0)	25.0 (0–500.0)	0.0 (900.0)
**Mean of overall fecal larvae reduction per group**		-------	91.4%	97.4%	89.9%	84.3%

Superscript letters (^a,b^) between columns indicate significant differences among groups at *p* < 0.05. Iv = Ivermectin, *Df* = *Duddingtonia flagrans*, *Ac* = *Artemisia cina* and *Df*/*Ac* = combination of *D. flagrans* with *A. cina*.

**Table 3:** Effectiveness of orally administered *Duddingtonia flagrans* chlamydospore aqueous suspension in reducing gastrointestinal parasitic infective larvae in ruminant fecal cultures.

Treatment	Nematode	Results	References
In lambs and kids (12–20 weeks old) At doses of 250,000 or 500,000 spores/kg live weight, administered on two consecutive days	*Haemonchus contortus, Ostertagia* (*Teladorsagia*) *circumcincta*, or *Trichostrongylus colubriformis*.	78% effectivity in fecal samples	[31]
In sheep, six treatments of 5 × 10^4^, 1 × 10^5^, 2.5 × 10^5^, 5 × 10^5^, and 1 × 10^6^ chlamydospores/kg of body weight for 7 days	Gastrointestinal nematodes Larvae in feces	The reduction in infective larvae ranged from 76.6 to 100.0%.	[32]
In lambs *D. flagrans* chlamydospore aqueous suspension, at 5 × 10^5^ chlamydospores/kg BW Orally administered every third day for 15 days	Gastrointestinal nematodes EPG and infective larvae from coprocultures	EPG Reduction = 62.6 % Larval reduction = 96.2%	[16]
In Cebu calves, 0.025 × 10^6^ 0.5 × 10^6^ 1 × 10^6^ *D. flagrans* chlamydospore aqueous suspension, every day for ten days	Gastrointestinal nematodes Larvae (L3) in feces	The highest larvae reduction = 88.5, 95.8, and 88.9%	[33]

**Table 4:** The effectiveness of the oral administration of *Artemisia* spp. extracts in reducing EPG values in small ruminants.

Extract	Percent Reduction in EPG or Larvae	Doses	Authors
*Artemisia cina* hexanic extract	76.6% of the larvae (in vitro)	4 mg/mL	[23]
*Artemisia cina hexanic* extract	31.7 % in EPG and 86.9% of the larvae (in vivo)	4 mg/mL	[38]
*Artemisia annua* ethanolic extract	24.7 % of the larvae (in vitro)	600 mg/kg	[39]
*Artemisia campestris* ethanolic extracts	91.3 % of the larvae (in vitro)	2 mg/mL	[40]

## Data Availability

Data are contained within the article.
